# Mesenchymal-to-Endothelial Transition in Kaposi Sarcoma: A Histogenetic Hypothesis Based on a Case Series and Literature Review

**DOI:** 10.1371/journal.pone.0071530

**Published:** 2013-08-06

**Authors:** Simona Gurzu, Diana Ciortea, Teodora Munteanu, Iringo Kezdi-Zaharia, Ioan Jung

**Affiliations:** 1 Department of Pathology, University of Medicine and Pharmacy of Tirgu-Mures, Tirgu-Mures, Romania; 2 Department of Dermatology, University of Medicine and Pharmacy of Tirgu-Mures, Tirgu-Mures, Romania; 3 Department of Infectious Diseases, University of Medicine and Pharmacy of Tirgu-Mures, Tirgu-Mures, Romania; University of Southern California Keck School of Medicine, United States of America

## Abstract

**Objectives:**

Although several studies have been conducted regarding Kaposi sarcoma (KS), its histogenesis still remains to be elucidated. The aim of our study was to analyze the immunophenotype of Kaposi sarcoma and to present a hypothesis about the histogenesis of this tumor, based on a case series and a review of relevant literature.

**Methods:**

In 15 cases of KSs diagnosed during 2000–2011, the clinicopathological features were correlated with the immunoexpression of c-Kit, SMA, CD34, CD31, vascular endothelial growth factor (VEGF), COX-2, c-KIT, smooth muscle antigen (SMA), and stem cell surface marker CD105.

**Results:**

Both CD105 and c-KIT rate of the spindle-shaped tumor cell positivity increased in parallel to the pathological stage. All cases displayed CD105 and weak c-KIT positivity in the endothelial cells. SMA, VEGF, and COX-2 were focally expressed in all cases. CD34 marked both endothelium and spindle-shaped tumor cells. No c-KIT expression was noticed in KS of the internal organs.

**Conclusions:**

KS seems to be a variant of myofibroblastic tumors that originates from the viral modified pluripotent mesenchymal cells of the connective tissue transformed in spindle-shaped KS cells, followed by a mesenchymal-endothelial transition and a myofibroblastic-like differentiation. This paper mailnly showed that KS cannot be considered a pure vascular tumor.

## Introduction

Kaposi sarcoma (KS) was first described in 1872 by Moritz Kaposi as an idiopathic hemorrhagic-pigmented sarcoma of the skin (“sarcoma idiopathicum multiplex hemorrhagicum”), which affects elderly male subjects [1]. Although seminal advancements have been made regarding the understanding of the tumor, its histogenesis is still controversial. Some authors still consider that KS is a low-grade vascular tumor associated either with either HIV infection or immunosuppression [2], [3]. An important step was performed in the understanding of its etiology, with the evidence of the relation between human herpes virus 8 (HHV-8) and KS [4]; HHV-8 can be detected in the patient's blood 5–10 years before occurrence of the clinical symptoms [5].

The immunohistochemical features of KS could also help in the elucidation of its histogenesis. To asses this goal, we analyzed the immunohistochemical expression of c-KIT, CD34, CD31, CD105, smooth muscle actin (SMA), vascular endothelial growth factor (VEGF), and COX-2 in KS cells and performed a review of the relevant literature related to these aspects.

C-KIT protein is encoded by the C-KIT gene located on chromosome 4q12 and plays an important role in the development of hematopoietic stem cells, mast cells, germ cells, melanocytes, and interstitial cells of Cajal [6]. Regarding the tumor cells, c-KIT (CD117) is positive in gastrointestinal stromal tumors, but overexpression in several mesenchymal tumors including melanoma, angiosarcoma, and KS was also reported [3], [6], [7].

CD34 is an endothelial marker that marks both normal, preexisting vessels and the neoformed intratumoral angiogenic-activated ones [8], [9]. This marker is also present in the thyroid interfollicular cells [10] and can be overexpressed in cancer cells, in tumors such as gastrointestinal stromal tumors, inflammatory fibroid polyp or myofibroblastoma [11], [12].

CD105 (endoglin) is a homodimeric transmembrane glycoprotein, a modulator of angiogenesis that marks the angiogenic tumor blood vessels but is not expressed by the normal preexisting mature large vessels [8], [9], [13]. To our knowledge, only one of the previously reported studies examined the CD105 expression in KS, but the authors declined its positivity in the tumor spindle cells [14].

SMA is a usual marker used for differential diagnosis of several tumors. Beside smooth muscle fibers, it also marks the fibroblasts and myofibroblasts being overexpressed in some mesenchymal tumors such as leiomyoma, leimyosarcoma, myofibroblastoma, inflammatory myofibroblastic tumor, and gastrointestinal stromal tumors with myogenic differentiation [11], [12], [15]. A slight expression of SMA was also reported in spindle-shaped KS cells [7], [16], but its significance was not elucidated yet.

VEGF is known to be a proangiogenic factor involved in physiological and pathological angiogenesis. Enzymes codified by the PTGS2 gene, the cyclooxygenase isoforms (COX-1 and COX-2 or prostaglandin-endoperoxide synthase 2) regulate the prostaglandin synthesis via arachidonic acid. COX-1 is expressed in most of the normal human tissues in physiological conditions. COX-2 is related to cellular stress response pathways, being inducibly overexpressed in inflammatory processes, but its secretion is also stimulated by oncogenes, cytokines, growth factors, tumor promoters, and hormones, being implicated in cellular proliferation, tumor growth, invasion and hematogenous metastasis [17], [18], [19]. No data about its expression in KS cells have been published.

## Materials and Methods

The clinicopathological features of KS were analyzed in all consecutive cases diagnosed in a period of eleven years (2000–2011). Processing of the cases was approved by the ethical committee of the University of Medicine and Pharmacy of Tirgu-Mures, Romania. The patients have submitted their informed consent form for the publication of their case details. Microscopically, KS of the skin was classified into three main types:

- Patch stage - characterized by vascular proliferation, sparse spindle cells, lymphocytes, and rare extravasated erythrocytes
- Plaque stage - characterized by association of slit-like spaces with proliferation of spindle cells, in the dermis and irregularly in the hypodermis
- Nodular/tumor stage - the dermis was totally replaced by nodular sarcomatous proliferation of spindle cells, which displayed hyaline globules, extravasated erythrocytes, and hemosiderin deposits

The immunohistochemical stains were performed on paraffin-embedded tissues, using the UltraVision System (LabVision, Fremont, CA, USA). For each case with a unique lesion a number of three to five slides were evaluated (cases 1, 3, 4, 7, 9, 12, 13, and 14; Table 1). For synchronous tumors, a number of three to five biopsies were performed and analyzed for each of the patients (cases 2, 5, 6, 8, 10, 11, and 15; Table 1). The antibodies CD31 (JC70A clone), CD34 (Qbend/10 clone), and c-KIT (polyclonal rabbit) from Dako (Glostrup, Denmark), diluted at 1∶200, 1∶100, and 1∶500, respectively, were used. SMA (1A4 clone), VEGF-A (clone VG1), and CD105 (SN6H clone) antibodies were diluted at 1∶600, 1∶25, and 1∶50, respectively (LabVision, Fremont, CA, USA). COX-2 (4H12 clone) was diluted at 1∶200 (Novocastra, Newcastle-upon-Tyne, UK). The prediluted monoclonal HHV-8 antibody (clone 13B10) was from MyBioSource (San Diego, CA, USA). The heat-induced antigen retrieval was performed in high-retrieval solution (LabVision) for c-KIT and VEGF antibodies. In case of CD31, CD34, HHV-8, CD105, COX-2, and SMA, pepsin-enzymatic retrieval (LabVision) was used. The slides were developed using DAB (diaminobenzidine) solution (Novocastra). Counterstaining was performed using Mayer's hematoxylin (Novocastra).

**Table 1:** Clinicopathological and immunohistochemical features of patients with Kaposi sarcoma (F = female; M = male; V = vessels; T = tumor cells; ± referes to focal positivity and + represents diffuse expression).

No. of case	Sex	Age (years)	Localization	Macroscopic feature	Microscopic type	c-KIT		CD105		SMA	VEGF		COX-2	
						V	T	V	T	T	V	T	V	T
1	M	65	skin - sole	single nodule	nodular	±	+	+	+	±	±	±	±	±
2	M	70	skin - sole	multiple plaques	patch	−	±	+	±	±	±	±	±	±
3	M	53	skin - thigh	single nodule	nodular	+	+	+	+	±	±	±	±	±
4	M	58	skin - shin	ulcerated tumor	patch	−	−	+	±	±	±	±	±	±
5	M	34	skin - auricle, forearm, thigh and shin	nodular protruded tumors	nodular	±	+	+	+	±	±	±	±	±
6	M	30	skin - multiple nodules on the limbs	nodular protruded tumors	nodular	±	+	+	+	±	±	±	±	±
7	M	75	skin - sole	single nodule	plaque	−	±	+	±	±	±	±	±	±
8	M	86	skin - left and right big toes	ulcerated tumors	plaque	±	±	+	±	±	±	±	±	±
9	M	42	pharynx	infiltrative tumor	nodular	−	−	+	±	±	±	±	±	±
10	M	76	skin - shin	multiple tumors	plaque	−	±	+	±	±	±	±	±	±
11	M	30	both tonsils	infiltrative tumors	nodular	−	−	+	±	±	±	±	±	±
12	M	48	stomach	protruded tumor	nodular	−	±	+	±	±	±	±	±	±
13	F	70	skin - shin	ulcerated tumor	patch	−	−	+	±	±	±	±	±	±
14	F	65	skin - shin	ulcerated tumor	nodular	±	+	+	+	±	±	±	±	±
15	F	20	skin – multiple macules	macules (flat patches)	patch	−	±	+	±	±	±	±	±	±

In case of positivity in more than 40% of spindle-shaped tumor cells, we considered the cases with diffuse positivity. Cases that displayed positivity in 10–40% of tumor cells were considered to have focal positivity; the cut-off value of positivity was 10%.

## Results

### Clinicopathological features

In a period of eleven years, 15 cases were diagnosed. Twelve male and three female subjects were included in this study. The median age of patients was 54.8±19.997 years (20–86 years). One case (case 15, Table 1) was diagnosed in a C3-AIDS-staged female and occurred 7 years after self-cessation of highly active antiretroviral therapy (HAART). Another case (case 5) occurred two years after complex radiochemotherapy performed for a metachronous testicular and thyroidian malignant tumors (case 5, Table 1); no significant medical history was reported in the other 13 cases (Table 1). All 15 patients are still alive without reported recurrences.

### Immunohistochemical aspects

- *CD31 and HHV-8 expression*

The endothelial cells of small and large vessels were marked by CD31 in all the 15 cases without positivity within the spindle-shaped tumor cells. Focal immunoexpression for HHV-8 was observed in the tumor cells, in all cases in correlation with high levels of serum HHV-8.

- *c-KIT expression*

Table 1 shows that the two cases localized in the tonsils (case 11) and the pharynx (case 9) were c-KIT negative. The gastric KS (case 12) presented a focal c-KIT expression in tumor cells, but the endothelial cells were negative. None of these three cases presented skin involvement.

**Figure 1:**
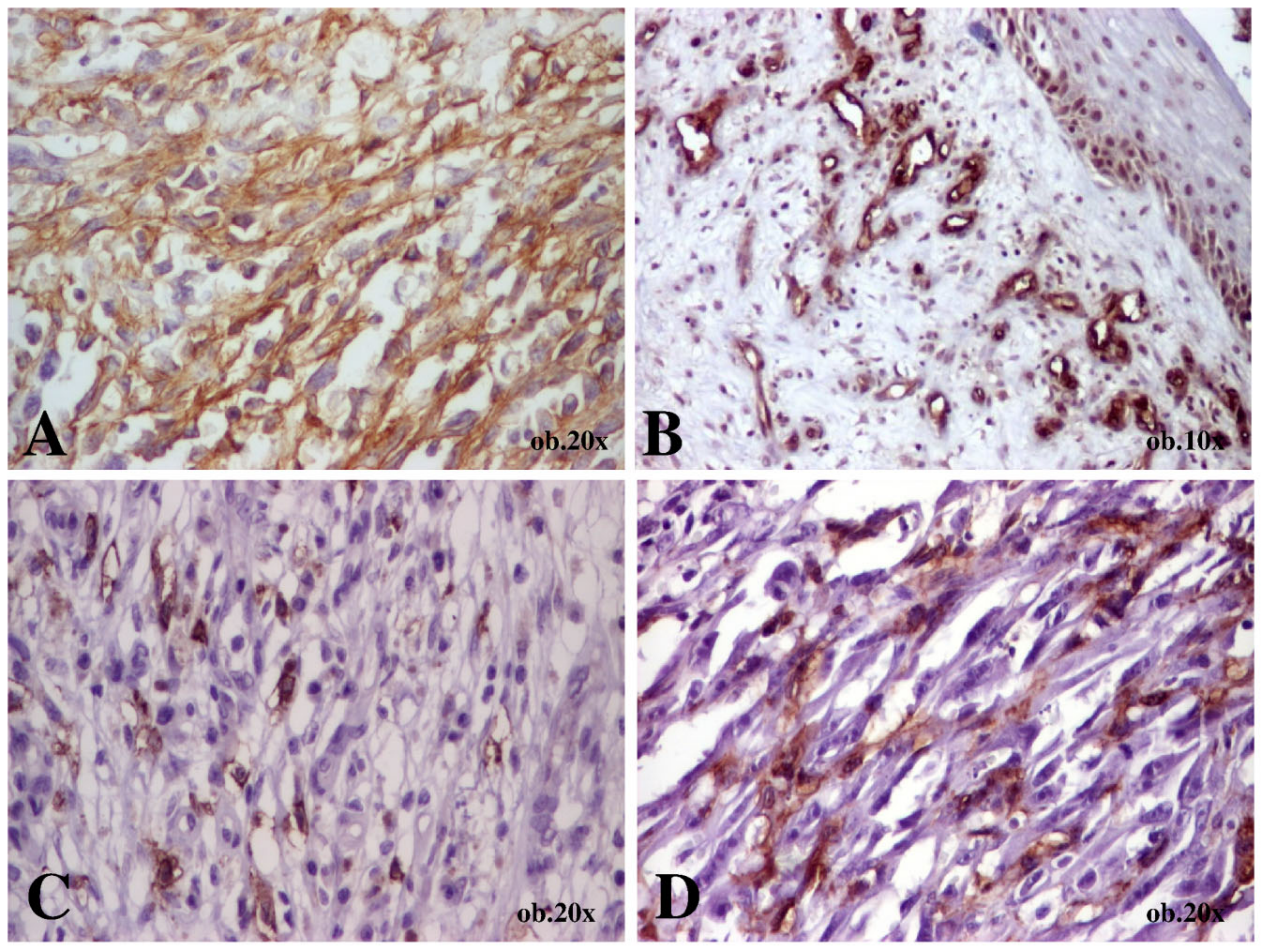
Immunohistochemical features of a plaque-stage Kaposi sarcoma diagnosed in a 75-year old male that presented a nodule on the sole (case 7). A- Diffuse expression of CD34; B - Endothelial positivity of CD105; C - Focal expression of c-KIT; D - Focal expression of SMA.

**Figure 2:**
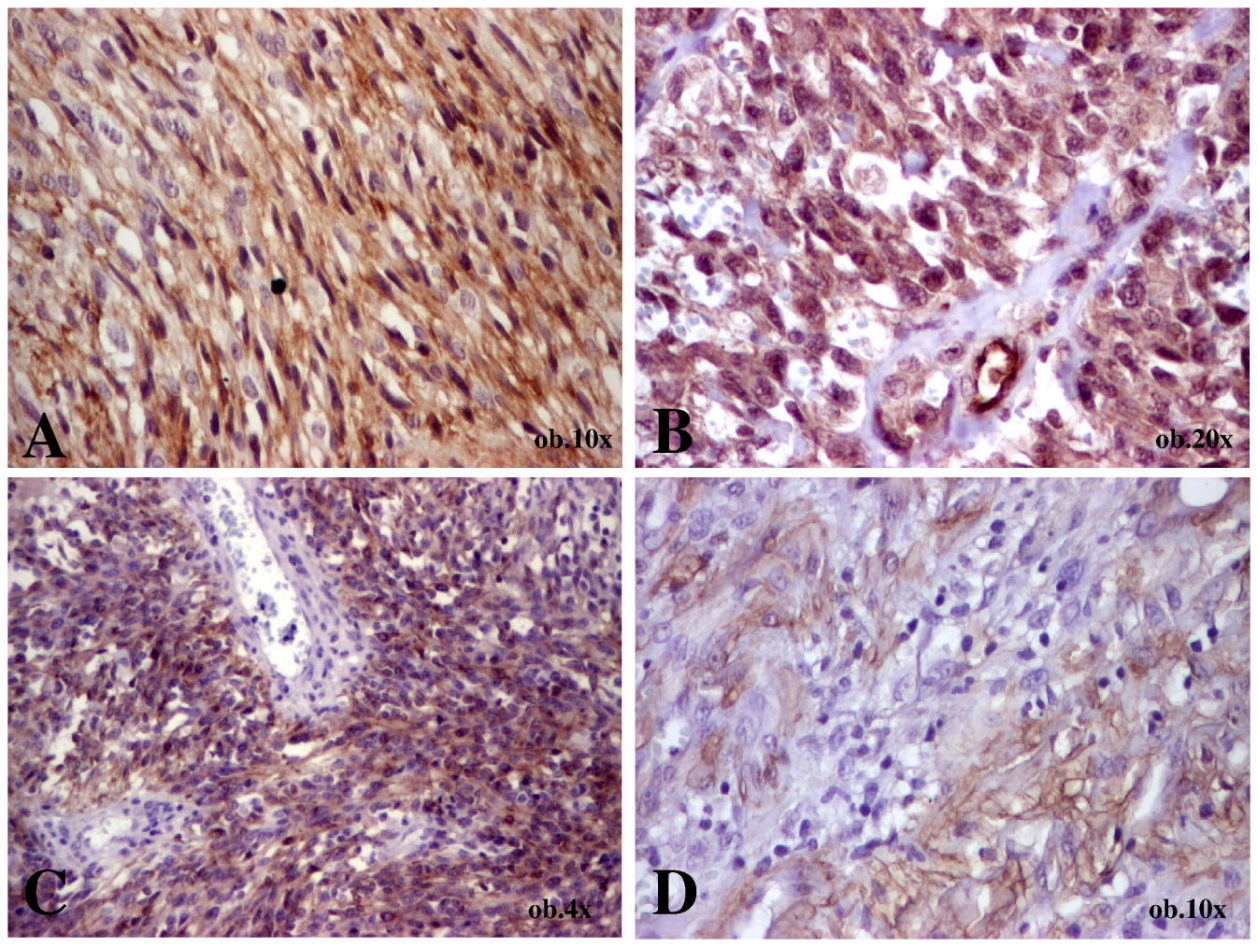
Immunohistochemical features of a nodular-stage Kaposi sarcoma diagnosed in a 65-year old male that presented a nodule on the sole (case 1). A- Diffuse expression of CD34; B – Diffuse expression of CD105 in the endothelial cells and spindle-shaped tumor cells; C - Diffuse expression of c-KIT in the spindle-shaped tumor-cells without endothelial positivity; D - Focal expression of SMA.

In the twelve KSs of the skin, the c-KIT positivity was correlated to the pathological stage of the tumor. In early stage, c-KIT was either absent (cases 4 and 13) or focally positive in the spindle cells (cases 2 and 15), with negative endothelium. In the plaque stage (cases 7, 8, and 10), c-KIT was focally observed in tumor cells and present or absent in the endothelium. However, in all nodular-type KSs (cases 1, 3, 5, 6, and 14), the tumor cells diffusely expressed c-KIT, and some of the intratumoral vessels were also positive (Figs. 1, and 2).

- *SMA and CD34 expression*

**Figure 3:**
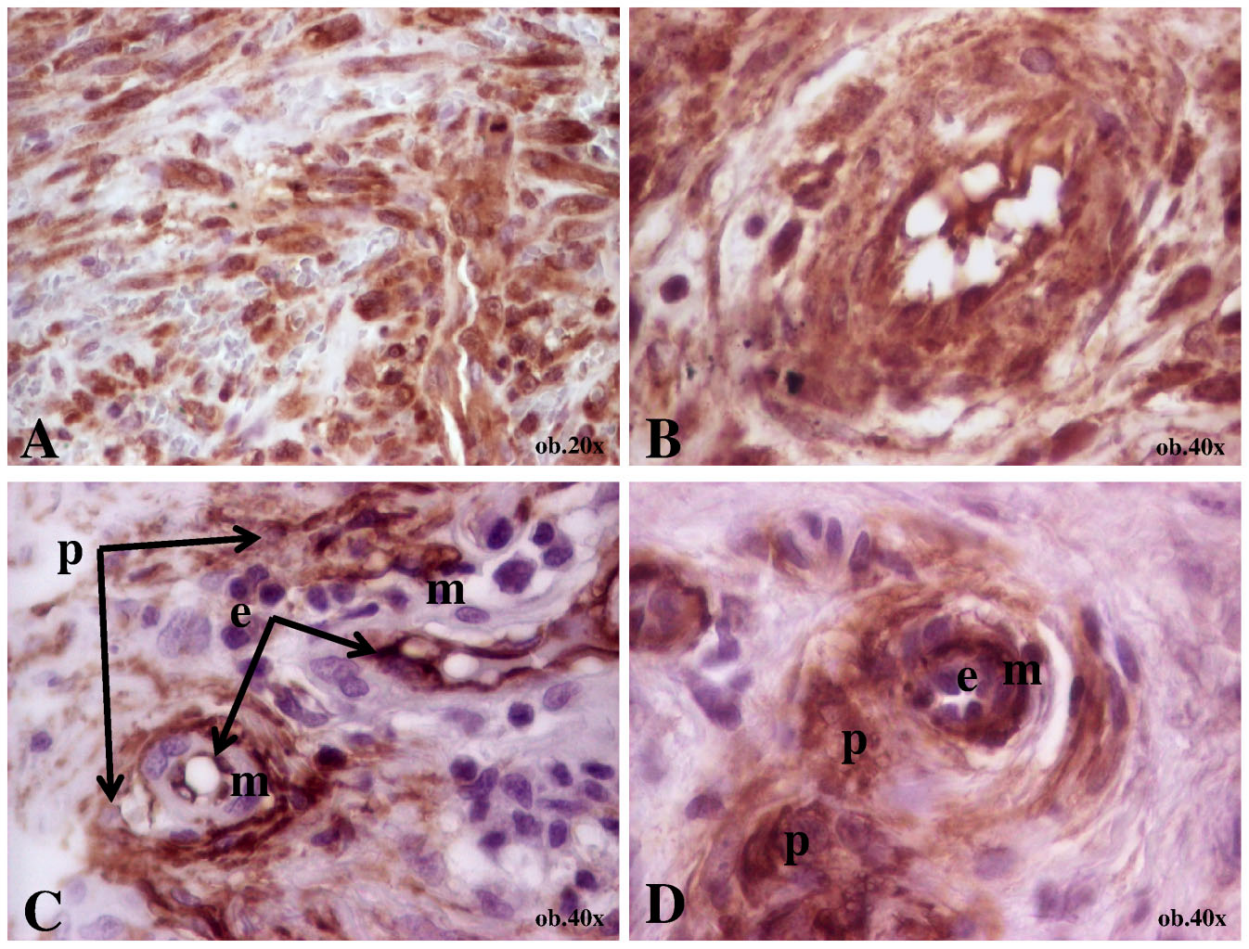
Immunohistochemical particularities of a nodular-stage Kaposi sarcoma diagnosed in a 53-year old male that presented a nodule on the thigh (case 3). A - Diffuse expression of COX-2; B – VEGF is diffusely expressed in the spindle-shaped tumor cells with focally slit-like leaky pseudovascular spaces; C - CD34 marks both endothelial cells (e) and the perivascular tumor cells (p) but the middle vascular layer (m) was negative; D - SMA expression can be seen in the middle vascular layer and the perivascular tumor cells.

Both CD34 and SMA presented either focal or diffuse expression in the tumor cells. The high-power view examination revealed a specific positivity of the large intratumoral vessels. CD34 usually marked the endothelial cells, the external vascular layer, and some of the perivascular cells. At the same time, SMA was negative in the inner endothelium but marked the middle vascular layer and some of the perivascular cells (Fig. 3). The endothelium of the large mature vessels, marked by CD34, was c-KIT negative (Fig. 2).

- *CD105 expression*

CD105 marked the vascular endothelium in both small and large intratumoral vessels, in all cases. Regarding the positivity of spindle-shaped tumor cells, no negative cases were identified. The non-skin tumors (cases 9, 11, and 12) presented focal positivity. In the tumor of the skin, CD105 expression was similar to c-Kit one (Table 1), being focally expressed in cases diagnosed in patch or plaque stages; diffuse positivity was displayed by all nodular-type KSs (Figs. 1, and 2).

- *VEGF and COX-2 expression*

In all cases, VEGF and COX-2 were focally expressed in both endothelial and spindle-shaped tumor cells. Focally, slit-like leaky spaces with extravasted-erythrocytes but without endothelial cells, marked by VEGF, were observed (Fig. 3).

## Discussion

Despite the *in vivo/in vitro* studies and also the complexity of immunohistochemical or molecular examinations, the histogenesis of KS remains controversial. Most authors maintain that KS is a HHV-8-related tumor of endothelial origin that can be treated using antiangiogenic substances [20], [21]. In contrast, our immunohistochemical staining results, suggest that KS originates from bone marrow mesenchymal stem cells (BM-MSC) derived from pluripotential mesenchymal stem cells of the connective tissue (PMC).

The BM-MSCs were first described in 1976 by Friedenstein *et al.* as a fibroblast-like cell population of the bone marrow (“marrow stromal cells”) [22], which express CD105, CD90, and CD73 antibodies. They are not hematopoietic cell derivatives because they do not display CD34, c-KIT, CD45, or CD14 [23], [24].

On the other hand, PMCs were identified in the connective tissue of the dermis, skeletal muscles, and other organs [10]. They can originate from BM-MSCs [23], [24], from fibroblasts reprogrammed into pluripotent cells [25], or from circulating fibrocytes [26], [27]. The PMCs express CD105, CD90, CD34 and SMA but are negative for c-KIT and also for CD3, CD4, and CD8 [23], [24], [26]–[31]. These cells could be infected by viral vectors and can present genetic abnormalities at the genetic and epigenetic level that can be related with some of the oncogenic pathways [31]–[36]. The genetically modified PMCs secrete cytokines, integrins, and growth factors including VEGF [37] and possess a multilineage capacity. Depending on the induction medium, PMC can be differentiated into mesodermal and neuroectodermal lineage [28]–[31]; adipocytes, osteoblasts, chondrocytes, smooth muscle cells, fibroblasts, neural-like cells, and endothelial cells can derive from PMCs [10], [11], [23], [29], [38]. The mesodermal lineage acquires c-KIT and CD34 positivity [31]. C-KIT positive spindle-shaped cells were also identified in the adult dermis, being in contact with blood capillaries and perifollicular sheaths, which seem to be involved in skin remodeling and regeneration [26]. These cells were presented in literature as dendrocytes [39], dendritic cells [40], [41], or telocytes [26], probably variants of PMCs.

The positivity of spindle-shaped KS tumor cells for CD105 and COX-2, first reported in our study, in correlation with CD34, VEGF, SMA, and c-KIT positivity [3], [7], [41], seems to suggest that KS, similar to other fibroblastic tumors, originates from the PMCs and is related to the VEGF secretion. To sustain the complex and heterogenic origin of KS, other authors revealed an overexpression of the proapoptotic marker bcl-2 that promotes survival and reduces apoptosis in endothelial cells [42]. Positivity for the lymphatic markers D2-40 and Lyve-1was also reported, suggesting a mixed lymphatic and vascular origin [43], [44]. However, whether it is about an endothelial-mesenchymal or mesenchymal-endothelial transition is unclear.

**Figure 4:**
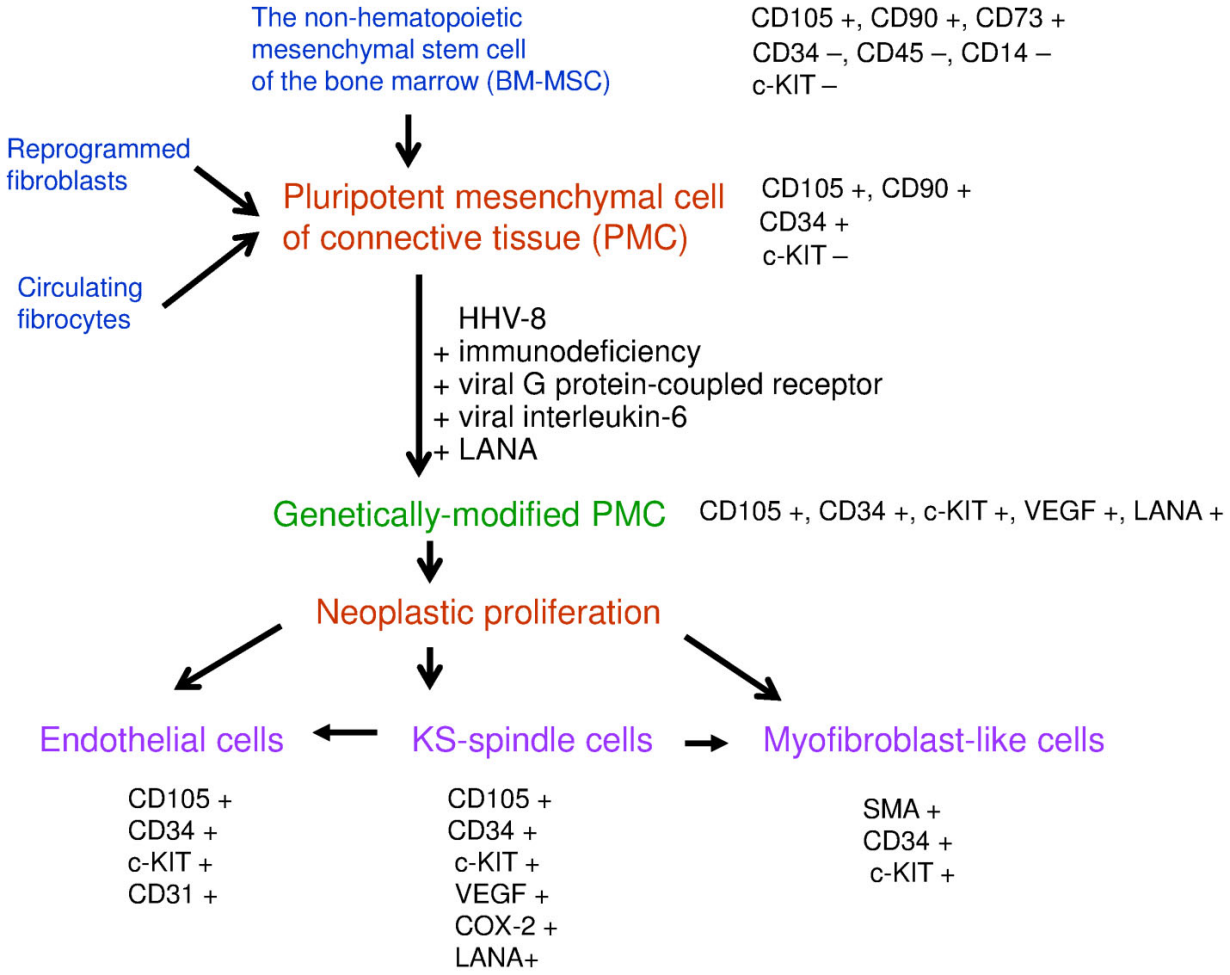
Histogenesis of Kaposi sarcoma: a hypothesis about viral-induced mesenchymal to endothelial transition, based on the immunohistochemical features of the precursor cells and the tumor cells (LANA = latency associated nuclear antigen).

On the one hand, the c-KIT endothelial positivity suggests a reversion of the tumor cells to fetal endothelial cell phenotype similar to angiosarcomas [6], [11], [15] and can sustain the origin of KS in the precursor circulating the pluripotent endothelial cells and also the possible endothelial-mesenchymal transition. At the same time, a literature synthesis performed by Uldrick *et al.* (2012) shows that in HHV-8-induced KS, the viral gene products, such as viral G protein-coupled receptor (vGPCR), viral interleukin-6, and latency-associated nuclear antigen (LANA), upregulate VEGF production [20] from the genetically modified PMCs [37], [45] but also promote cell proliferation, with vGPCR being sufficient to induce the angiogenic phenotype [42]. Based on these observations and our results, we hypothesis that the spindle-shaped KS cells derive from the genetically modified PMCs. The presence of HHV-8 in both PMCs [20], [32]–[36] and DNA of KS spindle cells [42] also argues for this origin. VEGF and COX-2 secretion from these cells leads to vascular differentiation, but SMA positivity also suggests a myofibroblast-like differentiation. This hypothesis, synthesized in Fig. 4, advocates rather a mesenchymal-endothelial transition than an endothelial-mesenchymal one. The mesenchymal-endothelial transition is also proven by the lower rate of responders to the antiangiogenic therapy in KS [20] compared with other vascular tumors such as angiosarcoma [46].

In line with our results, some studies revealed that c-KIT was expressed in KS but did not show a correlation with other clinicopathological factors [3], [7], and the CD105 positivity in the tumor cells was denied [14]. In our material, both CD105 and c-KIT expression in the spindle-shaped KS cells increased from early- to late-stage KS, proving their role in the evolution of KS. These data are in line with the results of Pyakurel *et al.* who reported an increasing number of LANA-positive cells from early- to late-stage KS [44]. A continuous recruitment of tumor precursor cells, probably PMCs, seems to be present during KS evolution, probably one of the reasons why long-term chemotherapy is often required in these patients [20].

This paper primarily aimed to show that KS cannot be considered a pure vascular tumor and seems to originate from the viral modified PMCs. A mesenchymal-endothelial transition and a myofibroblastic-like differentiation seem to be the key events of its origin. This hypothesis could influence the therapeuthical management of KS.
